# Indocyanine Green–Guided Pediatric Tumor Resection: Approach, Utility, and Challenges

**DOI:** 10.3389/fped.2021.689612

**Published:** 2021-09-20

**Authors:** Abdelhafeez Abdelhafeez, Lindsay Talbot, Andrew J. Murphy, Andrew M. Davidoff

**Affiliations:** Department of Surgery, St. Jude Children Research Hospital, Memphis, TN, United States

**Keywords:** indocyanine green, fluorescence, pediatric solid tumor, near infrared spectroscopy, intraoperative imaging

## Abstract

Incomplete tumor resection increases the risk of local recurrence. However, the standard of care approach to distinguishing tumor tissue is less than optimal, as it depends on a conglomeration of preoperative imaging and visual and tactile indicators in real time. This approach is associated with a significant risk of inadequate resection; therefore, a novel approach that delineates the accurate intraoperative definition of pediatric tumors is urgently needed. To date, there is no reliable method for the intraoperative assessment of tumor extent and real-time differentiation between tumor- involved tissues and tumor-free tissues. Use of intraoperative frozen sections is challenging, time consuming, and covers a small surface area. Increased vascular permeability and impaired lymphatic drainage in the tumor microenvironment leads to an enhanced permeability and retention effect of small molecules. ICG is a fluorescent dye that when administered intravenously accumulates passively in the tumor because of EPR, thereby providing some tumor contrast for intraoperative real-time tumor recognition. Preclinical and clinical studies suggest that the tumor-to-background fluorescence ratio is optimized when imaging is obtained 24 h after dye injection, and many studies suggest using a high dose of ICG to optimize dye retention in the tumor tissue. However, in childhood cancers, little is known about the ideal dosing, applications, and challenges of ICG-guided tumor resection. This retrospective study examines the feasibility of ICG-guided tumor resection in common childhood solid tumors such as neuroblastoma, sarcomas, hepatic tumors, pulmonary metastases, and other rare tumors. Pediatric dosing and challenges related to the optimization of tumor-to-background ratio are also examined.

## Introduction

Complete resection of solid tumors is critical for curing pediatric patients. The standard of care intraoperative approach to distinguishing tumor tissue depends on surgeons building a mental map of the tumor by using preoperative imaging and intraoperative visual and tactile indicators. However, this approach is associated with the risk of inadequate resection due to the lack of intraoperative real-time delineation of 3D tumor anatomy. The rate of local tumor recurrence for patients may be decreased and the survival increased by ensuring the completeness of resection by using fluorescence-guided surgery. Fluorescence-guided surgery uses a real-time tool for enhancing the visualization of the 3D tumor anatomy and facilitates differentiation between tumor and normal tissue.

Indocyanine Green (ICG) is a Food and Drug Administration (FDA)–approved water-soluble tricarbocyanine fluorophore that was initially used in clinical settings for measuring cardiac output, liver function, retinal angiography, and more recently for blood, biliary, and lymphatic flow imaging. ICG has been safely used in clinical studies for over 50 years at an FDA-approved pediatric maximum dose of 2 mg/kg IV. ICG is the most studied fluorophore, has a good safety profile, and is rarely associated with adverse reactions.

Many animal models have examined the utility of ICG-guided tumor localization, and more recently adult clinical trials have demonstrated the high sensitivity of this technique in identifying tumor cells (1–23). The difference in retention of ICG between tumor and normal tissue is the result of increased vascular permeability and impaired lymphatic drainage in the tumor microenvironment, which creates an enhanced permeability and retention (EPR) effect in small molecules (6).

Preclinical and clinical studies suggest that the tumor-to-background fluorescence ratio is optimized when imaging is obtained 24 h after injecting the intravenous dye (2, 21, 22), However, for liver primary tumors, a 72-h window has been suggested by others to allow greater washout of ICG from the adjacent normal liver tissue and improve the ability to identify tumors (24, 25). Adult trials have used 3–5 mg/kg ICG to optimize the tumor-to-background ratio, which is more than double the maximum FDA-approved pediatric dose. However, the necessity and safety of higher doses in children has not yet been studied. Moreover, the tumor biology of childhood tumors is different from that of adult tumors and ICG plasma clearance is significantly greater compared to adults (26). The aim of this study is to retrospectively examine ICG-guided tumor resection applications, dosing, and outcomes in pediatric oncology patients.

## Methods

This study was approved by the St. Jude institutional review board and waiver of informed consent was approved. We retrospectively reviewed the charts of all patients who underwent fluorescence-guided tumor resection at our institution from 2019 to 2020. Data on preoperative diagnosis and imaging findings, ICG dose, timing of ICG administration, operative notes, Iridium system (Visionsense Corp, Philadelphia, PA) video recording, intraoperative fluorescence, background noise, histopathology report, and complications were collected. All patients received a 1.5 mg/kg of ICG intravenous infusion over 15 min the day before surgery, except for two patients with hepatoblastoma who received 1.5 mg/kg of ICG 72 h before surgery. Intraoperative visualization was conducted with the Iridium system optimized to detect ICG. The Iridium system provides excitation light at 805 nm, causing ICG to emit bands between 825 and 850 nm that are captured by a near infrared (NIR) camera. This system generates a real-time fused image of surgical anatomy by allowing the capture of the normal white light image simultaneously with the ICG fluorescence image. The fused images enable the surgeon to proceed with tumor resection guided by fluorescing tumor dimensions without the need to switch off the normal white light view necessary for field visualization and to safely conduct tumor resection.

Measurements of the primary tumor as well as the surrounding tissue were completed to determine if the tumor was consistently fluorescent compared to the background normal tissue adjacent to the tumor. Background noise was defined as persistent fluorescence of nearby normal organs.

Histology is the gold standard for determining the presence of tumor. Thus, the diagnostic test ICG was compared to the final pathology report to calculate true positives, true negatives, sensitivity, and specificity.

## Results

Fifty-five patients (28 males and 27 females; median age 10 years [range <1–21 years]) underwent fluorescence-guided tumor resection. Of them, eight underwent two procedures and one patient underwent three procedures. The total number of procedures done was 65, including 37 thoracic, 19 abdominal (other than nephron-sparing resections) and nine trunk and extremity operations (Table 1). Cancer was confirmed by histology in 52 procedures (80%), and no malignant tumors were found in 13 procedures (20%). The 13 procedures in which lesions were found other than tumors included 10 pulmonary wedge resections that were histologically confirmed to be the following: four lymph nodes, two granulomas, one nodular pulmonary ossification, one dystrophic calcification, one histoplasma, and one post-therapy Wilms tumor with no morphologic evidence of viable tumor. The three abdominal biopsies that yielded diagnoses other than tumors were confirmed by histology to be the following: one lymph node with sinus histiocytosis, one granuloma, and one reactive fibroblastic proliferation.

**Table 1:** Tumor localization with ICG: utility, dosing, dosing interval, and challenges.

**Procedures** ***n* = 65**	**Fluorescent tumors (true positive)** ***n* = 46**	**Fluorescent nodules but not tumors (false positive)** ***n* = 3**	**Nonfluorescent nodules but not tumors (true negative)** ***n = 10***	**Nonfluorescent tumors (false negative)** ***n = 6***	**Background noise**	**Source of background noise (for open vs. MIS)**	**Fluorescence-guided identification of tumors not detected by standard of care**
Thorax *n* = 37 (pulmonary lesions = 36, Mediastinal NB = 1)	24 (OS = 9, HB = 5, HCC = 1, ES = 3, NB = 2, NRSTS = 2, RMS = 1, CB = 1)	2 (Histoplasma = 1, reactive lymph node = 1)	8 (3 lymph nodes = 3, granulomas = 2, nodular pulmonary ossification = 1, dystrophic calcification = 1, post-therapy Wilms tumor with no morphologic evidence of viable tumor = 1)	3 (OS = 2, WT = 1)	Total: 15 (40%) Open (11): 11(100%) MIS (26): 4 (15%)	Open: Skin, diaphragm, and chest wall MIS: Diaphragm, chest wall	3 lesions seen only by NIR (HB = 2, HCC = 1)
Abdomen *n* = 19	13 (HB = 3; HCC = 1, NB = 4, GCT = 2, NRSTS = 1, lymphoma = 1, SPNP = 1	1 (granuloma)	2 (Reactive fibroblastic proliferation = 1, lymph nodes with sinus histiocytosis = 1)	3 (ACC = 2, NB = 1)	Total: 13 (68%) Open (8): 8 (100%) MIS (11): 5 (45%)	Open: Skin, bowel, kidney, gall bladder MIS: Kidney, bowel	None
Trunk and extremities *n* = 9	9 (NRSTS = 4 RMS = 4 Myoepithelial carcinoma = 1)	0	0	0	9 (100%)	Skin	1 (Tumor extension seen only by NIR)

*ICG, Indocyanine Green; MIS, minimally invasive; OS, osteosarcoma; HB, hepatoblastoma; ES, Ewing sarcoma; RMS, rhabdomyosarcoma; NRSTS, non-rhabdomyosarcoma soft tissue sarcoma; ATC, anaplastic thyroid cancer; CB, chondroblastoma; NB, neuroblastoma; HCC, hepatocellular carcinoma; WT, Wilms tumor; GCT, germ cell tumor, SPNP, solid pseudopapillary neoplasm of pancreas; ACT, adrenocortical tumor*.

Of the 52 procedures that confirmed tumors, 46 (88%) were identified with NIR guidance, while of the 13 nonmalignant lesions only three (23%) lesions were fluorescent. The 46 fluorescent tumors included nine hepatoblastomas (HBs) and two hepatocellular carcinomas (HCCs), nine osteosarcomas (OSs), six neuroblastomas (NBs), six non-rhabdomyosarcoma soft tissue sarcomas (NRSTSs), five rhabdomyosarcomas (RMSs), three Ewing sarcomas (ESs), two germ cells tumors (GCTs), one chondroblastoma (CB), one solid pseudopapillary neoplasm of the pancreas (SPNP), one lymphoma, and one myoepithelial carcinoma of the chest wall (Table 1).

The sensitivity of NIR to identify tumor was 88% and specificity was 77% (Table 2). NIR imaging could not identify two primary adrenocortical tumors (ACTs). NIR imaging also could not detect one retroperitoneal metastatic lymph node and three pulmonary metastases, including two osteosarcoma metastases and one Wilms tumor metastasis. Two of these pulmonary metastases were small (<0.5 cm) and subpleural.

**Table 2:** Sensitivity and specificity of ICG-guided tumor localization.

**Sensitivity**	88%
**Specificity**	77%
**Positive predictive value**	94%
**Negative predictive value**	63%
**Accuracy**	86%

*ICG, Indocyanine Green*.

Background noise from adjacent organs was observed during a total of 37 procedures (57%), including all trunk and extremity resections, 68% of abdominal procedures (13), and 40% of thoracic procedures (15). Interestingly, background noise from adjacent organs was observed in all open abdominal and open thoracic procedures; however, this was seen in only 45% of minimally invasive (MIS) abdominal procedures (11) and only 15% of thoracoscopies (4) (Table 1).

**Figure 1:**
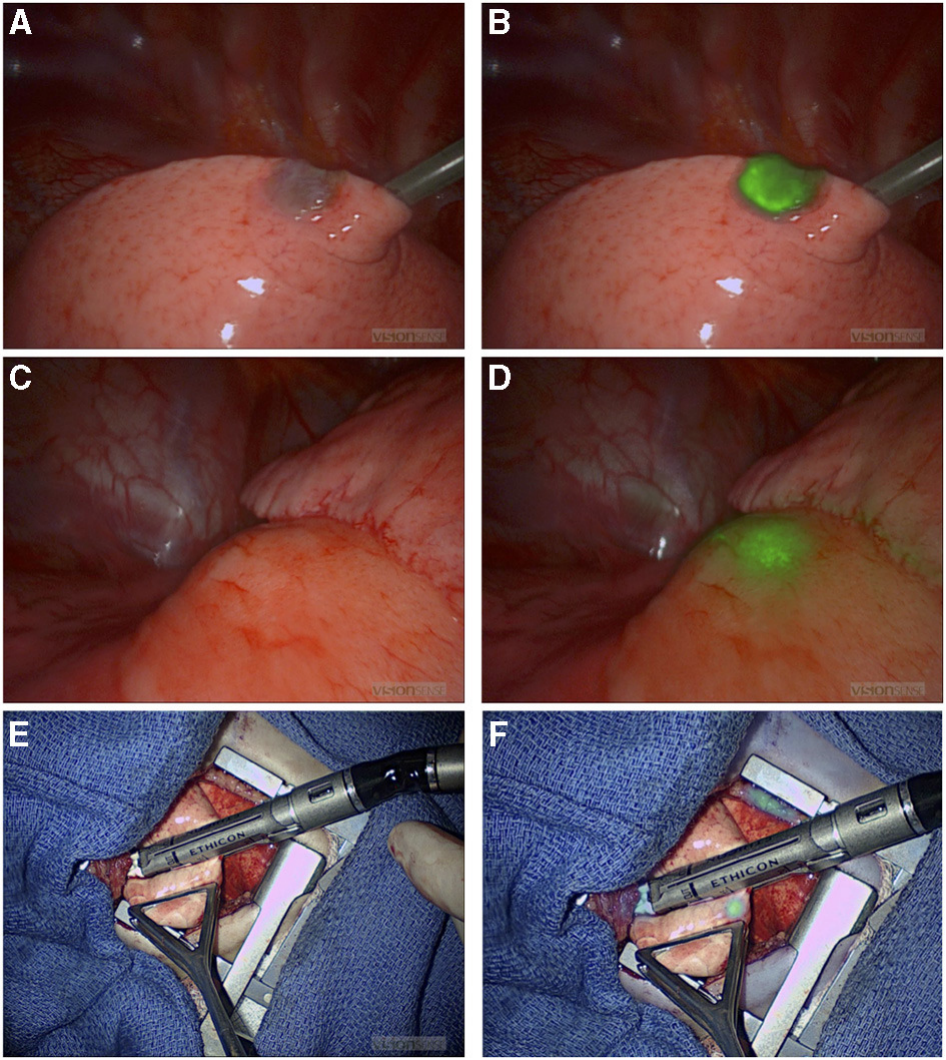
NIR-guided localization of pulmonary metastases for three thoracoscopic resections from a hepatic primary tumor. **(A,B)** NIR localization of a superficial nodule. This nodule was seen with both standard of care white light **(A)** and NIR **(B)**. NIR, near infrared. **(C,D)** NIR localization of a 2 cm nodule seen on the preoperative CT scan, but not visible when seen by standard of care white light **(C)**. **(D)** The same deep nodule was localized with NIR. NIR, near infrared; CT, computed tomography. **(E,F)** A small 0.2 cm nodule not localized with preoperative CT scan or with standard of care white light/tactile feedback **(E)**. **(F)** The same nodule localized by NIR. NIR, near infrared.

**Figure 2:**
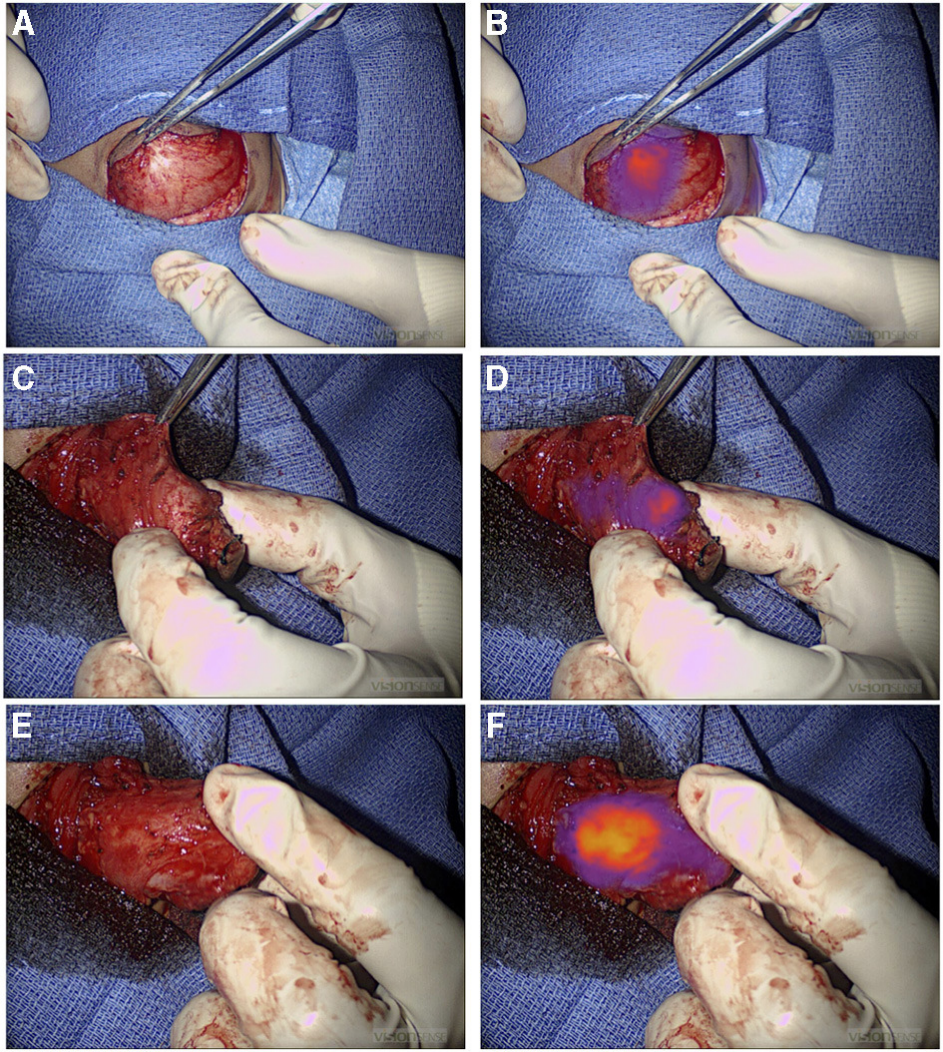
NIR guide localization of margin extension. **(A,B)** Initial view of a chest wall myoepithelial carcinoma with white light **(A)** and NIR **(B)**. NIR, near infrared. **(C–F)** Medial extension of the myoepithelial carcinoma was not appreciated with standard of care white light and tactile feedback **(C)**, but it was recognized by NIR **(D)**. Medial extension of the tumor was further localized with NIR after medial dissection proceeded **(E)** and **(F)**. NIR, near infrared.

NIR fluorescence provided localization guidance for three thoracoscopic resections of lung metastases of liver primary tumors (two HBs and one HCC) that were otherwise not seen by standard of care white light (Figure 1). NIR also provided surgical guidance for complete resection of a chest wall myoepithelial carcinoma with a medial tumor extension not differentiated from normal muscles with standard of care white light and tactile feedback (Figure 2). No adverse reactions were noted after administration of 1.5 mg/kg ICG in this study. Surgical complication rate was low in this cohort (5%), two patients developed postoperative seroma resolved after drainage and one patient developed air leak after pulmonary wedge resection responded to drainage.

## Discussion

We show that an ICG dose of 1.5 mg/kg is safe in pediatric patients receiving therapy for different types of malignancies. By using the study ICG dose and timing of injection, a broad range of pediatric malignant tumors consistently exhibited fluorescence compared to background tissue. These tumors included liver tumors, osteosarcomas, non-rhabdomyosarcomas, rhabdomyosarcomas, neuroblastomas, Ewing sarcoma, germ cells tumors, chondroblastoma, solid pseudopapillary neoplasms of the pancreas, lymphoma, and myoepithelial carcinoma. The sensitivity and specificity of ICG in identifying malignant tumor tissue intraoperatively were 88 and 77%, respectively. Moreover, the use of ICG in this study resulted in identifying 4 (6%) malignant lesions that would not have been identified by the standard of care.

Iridium systems examine large surfaces in real time with overlay of NIR images over standard white light visualization of the surgical field. Therefore, this system enables real-time fluorescence-guided tumor resection that allows the surgeon to maintain the principles of safe dissection without eliminating white light. At a dose less than the maximum FDA-approved pediatric dose, ICG accumulates in most pediatric solid tumors and is cleared from normal tissue within 24 h after injection. This was evident by the higher emission of photons from the tumor than adjacent organs, resulting in NIR imaging contrast demarcation between background tissue and most histology-confirmed tumors. The low specificity precludes strict interpretation of any fluorescence as tumor; hence, the risk of additional resection should be carefully weighed against the limitation of ICG-guided tumor bed assessment. Considering the high sensitivity of NIR imagery, the low specificity may not be a very significant limiting factor as the goal of most tumor surgeries is to avoid missing tumor deposits.

The more effective clearance of ICG in children from normal tissue may explain how optimal tumor-to-background ratio is achieved for NIR visualization of tumors with the ICG dose used in this study. Although, the incidence of background noise was high in open procedures likely as a result of ambient light contamination, this rarely limited the ability to delineate the tumor, as the source of background noise was mostly from organs and not directly adjacent to tumors such as bowel or skin tumors. Occasionally, organs adjacent to the tumor were the source of background noise and may have contributed to the negative fluorescence appearance of two ACTs, one of the two cases of osteosarcoma pulmonary metastasis, and one retroperitoneal metastatic lymph node relative to the intense signal from the kidney, diaphragm, and bowel, respectively. Proximity of the kidney did not preclude the optimal fluorescence of all included primary adrenal NBs (four patients). Moreover, ACT may require a different dosing or timing of ICG injection specific to this tumor biology for optimal imaging (22, 27). Measures to decrease ambient light contamination of the NIR field may mitigate background noise; however, it is unknown if further reduction in the ICG dose would reduce background noise without decreasing tumor signal and potentially diminishing the tumor-to-background ratio for some tumors. Another limitation of NIR fluorescence is tissue attenuation, which reduces photon count and precludes tissue penetration beyond a depth of 2 cm. The limitation of depth of penetration can potentially be resolved by optimizing filtering to reduce background noise and increase camera integration time. Two patients with small (<0.5 cm), subpleural pulmonary metastatic nodules required simultaneous guide-wire localization, as NIR imaging could not detect deep small lesions; therefore, it is advisable to use an alternative localization technique in similar scenarios and not rely merely on NIR imagery for small subpleural lesions.

**Figure 3:**
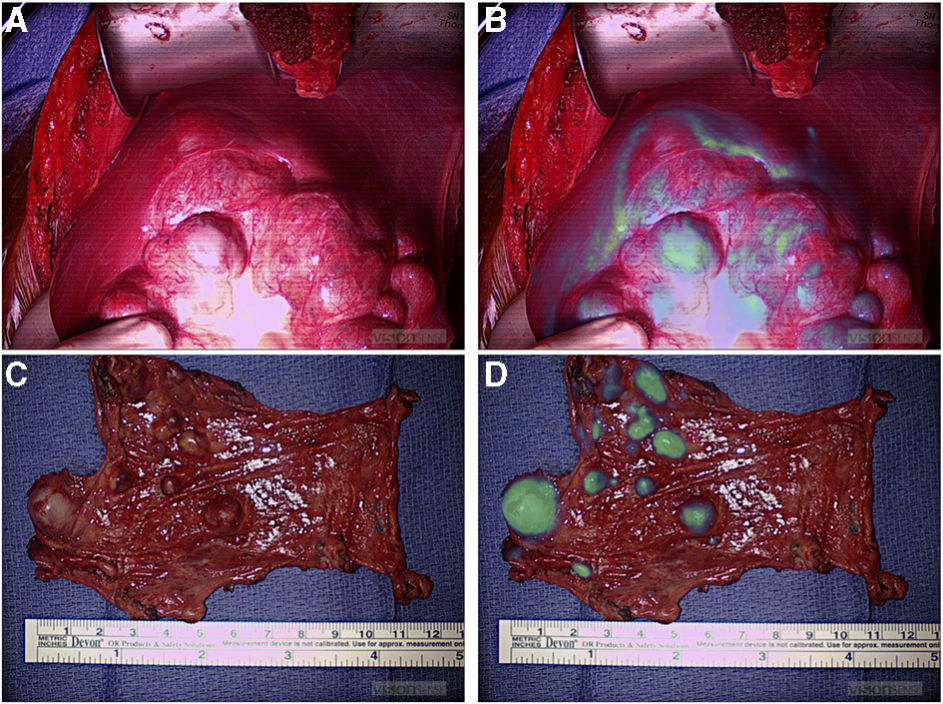
NIR localization of primary and metastatic liver tumors. **(A,B)** White light **(A)** and NIR **(B)** view of a primary liver hepatoblastoma. **(C,D)** White light **(C)** and NIR **(D)** view of hepatocellular carcinoma peritoneal metastases. NIR, near infrared.

Most tumor resections were amenable to localization with standard of care white light visualization and tactile feedback (94%). During four tumor resections (6%), ICG fluorescence provided surgical guidance to localize tumors otherwise not adequately localized by the standard of care. Three of these resections were pulmonary metastases from hepatic primary tumors appreciated only with ICG guidance. Although, one of these lesions was a 2 cm nodule seen on the preoperative CT scan, the depth of this nodule was 2.3 cm from the pleural surface, and it was not seen by intraoperative white light. The other two nodules were subcentimetric and neither was seen on the preoperative CT scan or with intraoperative standard of care. The utility of ICG hepatic tumor localization has been confirmed by previous studies (28, 29), which is in keeping with our findings that liver primary and metastatic deposits were consistently fluorescent (Figure 3). Also, ICG guidance helped identify a medial tumor extension of myoepithelial carcinoma of the chest wall otherwise not differentiated from adjacent normal muscle. This enabled achieving complete resection; however, it may not be possible to conclude on the utility of fluorescence-guided identification of tumor margins outside of prospective trials. We are currently prospectively examining the utility of ICG-mediated NIR imagery to discern tumor margins, identify residual disease, and study ICG uptake in pretreated tumors (30).

## Conclusion

ICG-guided tumor localization is a feasible adjunct for most pediatric solid tumors at a relatively lower dose than what is recommended in preclinical studies and adult clinical trials. ICG is highly sensitive for tumor tissue but its specificity is low. For deep and small lesions, an alternative localization technique needs to be used simultaneously that helps in cases where NIR cannot penetrate deep enough to identify a lesion. Background noise is less during the MIS approach, and optimal control of ambient light contamination may help mitigate this issue.

## Data Availability Statement

The original contributions presented in the study are included in the article/supplementary material, further inquiries can be directed to the corresponding author.

## Ethics Statement

The studies involving human participants were reviewed and approved by St. Jude Institutional Review Boards (IRBs), St. Jude Children's Research Hospital, Memphis, USA. Written informed consent from the participants' legal guardian/next of kin was not required to participate in this study in accordance with the national legislation and the institutional requirements.

## Author Contributions

All authors took part in writing the manuscript, reviewing it, and revising its intellectual and technical content. All authors assume responsibility and accountability for the results.

## Conflict of Interest

The authors declare that the research was conducted in the absence of any commercial or financial relationships that could be construed as a potential conflict of interest. The reviewer TL declared a past co-authorship with one of the authors AD to the handling editor.

## Publisher's Note

All claims expressed in this article are solely those of the authors and do not necessarily represent those of their affiliated organizations, or those of the publisher, the editors and the reviewers. Any product that may be evaluated in this article, or claim that may be made by its manufacturer, is not guaranteed or endorsed by the publisher.
